# Introducing biomarkers for invasive fungal disease in haemato-oncology patients: a single-centre experience

**DOI:** 10.1099/jmm.0.001564

**Published:** 2022-07-12

**Authors:** Anthony W. Martinelli, Callum B. Wright, Marta S. Lopes, Rosemary L. Swayne, Pramila Krishnamurthy, Charles Crawley, Ben Uttenthal, George Follows, Judith Babar, Sani H. Aliyu, David A. Enoch, Clare R. Sander

**Affiliations:** 1 Department of Respiratory Medicine, Cambridge University Hospitals NHS Foundation Trust; 2 Department of Haematology, Cambridge University Hospitals NHS Foundation Trust; 4 Department of Radiology, Cambridge University Hospitals NHS Foundation Trust; 5 Clinical Microbiology & Public Health Laboratory, UK Health Security Agency, Cambridge University Hospitals NHS Foundation Trust

**Keywords:** Aspergillosis, galactomannan, biomarkers, fungal infection, antifungal agents

## Abstract

**Objectives:**

Biomarkers for invasive fungal disease (IFD) have been shown to reduce antifungal prescriptions in neutropaenic haemato-oncology patients. Our study aimed to assess the real-life impacts of introducing a novel biomarker-based pathway, incorporating serum galactomannan and Aspergillus PCR, for pyrexial haemato-oncology admissions.

**Methods:**

Patients with neutropaenic fever were identified prospectively after introduction of the new pathway from 2013-2015. A historical group of neutropaenic patients who had blood cultures taken from 2009-2012 was generated for comparison. Clinical details including demographics, underlying diagnosis, investigations, radiology and antimicrobial treatment were obtained.

**Results:**

Prospective data from 308 patients was compared to retrospective data from 302 patients. The proportion of patients prescribed an antifungal medication was unchanged by the pathway (p=0.79), but the pattern was different with more patients receiving targeted antifungals (p=0.04). A negative serum galactomannan test was not sufficient evidence to withhold therapy with 17.2% of those episodes felt to have possible or probable IFD by EORTC/MSG criteria. There was no difference in 30-day mortality (p=0.21) or 1-year mortality (p=0.57) following introduction of the pathway.

**Conclusions:**

Biomarkers can be used safely as part of a multidisciplinary approach to the diagnosis of IFD in neutropaenic haemato-oncology patients. Whilst they do not necessarily result in antifungal therapy being withheld, they can allow more confident diagnosis of IFD and more specific antifungal therapy in selected cases.

## Introduction

Neutropaenic haemato-oncology patients are known to be at risk of invasive fungal disease (IFD), which is associated with high mortality rates, ranging from 10% to 57% at 90 days, depending on the subpopulation.(1–3) Whilst early diagnosis and treatment with appropriate antifungal therapies can improve outcomes, histological or microbiological confirmation of the infection is usually not feasible.(4) Therefore, clinicians use radiological evidence and, more recently, biomarkers to guide treatment.

The EORTC/MSG guidelines, published in 2008 and updated in 2019, provide standardized criteria for researchers to use in the diagnosis and classification of IFD.(5,6) These guidelines classify an IFD diagnosis as “possible” if a host factor indicative of susceptibility (e.g. haematological malignancy) is present alongside a clinical feature of IFD (i.e. classical radiological patterns). If additional mycological evidence is present - for example, culture of a mould from samples taken at bronchoscopy - the case can be further classified as “probable”. Galactomannan, a polysaccharide present in the cell wall of Aspergillus which can be measured in serum and bronchoalveolar lavage (BAL), was included as a mycological criterion for diagnosis of Invasive Aspergillosis (IA) in both 2008 and 2019, whilst PCR for Aspergillus was included in the latter edition only. Rarely, a diagnosis of IFD can be classed as “proven” by methods including visualization of invasive fungi in tissue samples from normally sterile sites or by growth of molds or yeasts in blood cultures.

Given that antifungal stewardship (AFS) can improve patient care and reduce costs, it is important to establish whether biomarkers can help guide therapy for presumed IFD.(7) Previously, it has been shown that biomarkers can allow empirical antifungal medications to be withheld safely when used as part of a clinical pathway for high-risk haemato-oncology patients admitted to hospital and that this is cost-effective.(8–10) A randomised controlled trial also found that, when compared to a standard diagnostic strategy of culture and histology, a biomarker-based approach reduced empirical antifungal treatment.(11) Nonetheless, the real-life impacts of increased biomarker testing have yet to be fully examined and we have previously shown that, in our tertiary hospital, use of BAL galactomannan resulted in overtreatment of some non-haemato-oncology patients, leading to drug-related toxicities.(12)

We sought to establish whether introducing a new pathway for neutropaenic fever, incorporating serum galactomannan testing and Aspergillus PCR, changed the frequency and patterns of antimicrobial prescriptions in a tertiary hospital. Secondary aims included establishing whether a negative serum galactomannan test was clinically useful. Our primary endpoints were the number of patients prescribed one or more antifungal medications at a therapeutic dose during their admission and the pattern of these prescriptions, particularly the proportion of patients where there was narrowing of the therapeutic spectrum. Secondary endpoints included whether the new pathway was non-inferior in terms of mortality and whether a negative serum galactomannan altered therapeutic strategy.

## Methods

Cambridge University Hospitals NHS Foundation Trust is a British Committee for Standards in Haematology level 3 referral hospital offering outpatient and inpatient haemato-oncology services, including haematopoietic stem cell transplantation.(13) In 2013, a new management pathway to be followed by non-consultant doctors admitting haematology patients with neutropaenic fever was introduced. The pathway included serum galactomannan testing on day 1, with serum Aspergillus PCR, repeat serum galactomannan and CT-thorax (reviewed by a specialist chest radiologist) on day 3 for patients where fever was not resolving (Supplementary Figure 1). Bronchoscopy was included within the pathway for appropriate patients with abnormal radiology, with samples taken including BAL galactomannan, BAL Aspergillus PCR, and culture. For fevers persisting after 72 hours, initial antifungal therapy with liposomal amphotericin B (L-Amb, Ambisome) was advocated as part of the pathway. All prescriptions were ultimately at the discretion of the treating consultant haematologist.

As part of a service evaluation following introduction of the pathway, patients with neutropaenic fever were identified prospectively from admissions to the hospital’s haemato-oncology ward from 08/04/2013 to 17/04/2015. A comparison retrospective data set was obtained by identifying neutropaenic patients who had blood cultures taken between 01/10/2009 and 30/04/2012. Clinical details including demographics, underlying diagnosis, laboratory investigations, radiology and antimicrobial treatment were obtained. Individuals admitted with pyrexia and neutropaenia on separate occasions were included once for demographic analysis, but each admission was considered as a distinct episode for analyzing management and outcomes. The 2008 EORTC/MSG guidelines were used to make a retrospective diagnosis of IFD per the published criteria.(5) Across both periods Trust guidance for antifungal prophylaxis was unchanged, advising the use of fluconazole where indicated.

Galactomannan analysis was performed using the BioRad platform (Bio-Rad, Hercules, CA, USA) according to the manufacturer’s instructions with a threshold ODI value of ≥0.500 (for both serum and BAL) to maximize sensitivity of the assay. Each positive sample was re-tested as specified in the kit insert. PCR was performed at the Public Health England Mycology Reference Laboratory.

Analysis of the first year of retrospective data from May 2011 to May 2012 estimated the 1-year mortality rate at 45% meaning the number of patients needed per group to establish non-inferiority was calculated as 298 (20% one-sided significance level, 80% power, inferiority margin of 5%). Although in this study mortality was a secondary outcome measure, this analysis was performed due to clinician concern that increased biomarker use would result in reduced antifungal prescription leading to increased mortality. Comparisons were performed using the chi-square calculator at www.icalcu.com and significance was taken as p ≤ 0.05.

## Results

In total, 302 patients were identified prior to the institution of the pathway and 308 patients after introduction of the pathway. The two cohorts were well matched in terms of sex, age, haematological diagnosis and transplant status (Table 1). Statistical analysis using a Cox proportional hazards model showed that potential risk factors for overall mortality at one year were comparable between the two cohorts, with allogeneic stem cell transplantation being a notable risk factor for increased mortality in both groups (Supplementary Figure 2).

The prospective cohort included 482 patient episodes, of which 352 (73.0%) included serum galactomannan testing (Tables 2 and 3). Of this cohort, 157 of 482 episodes (32.6%) included two distinct serum galactomannan results, and 102 episodes (21.1%) included serum Aspergillus PCR. Differing management strategies for patients categorized by their serum galactomannan result is detailed in Table 3. Serum Aspergillus PCR was positive in three episodes: one also included positive serum galactomannan, BAL galactomannan and BAL Aspergillus PCR and was therefore felt to represent probable IFD, whilst the other two episodes did not have radiological changes compatible with IFD or a second consecutive positive PCR, and were therefore treated as false positive results.

**Figure 1:**
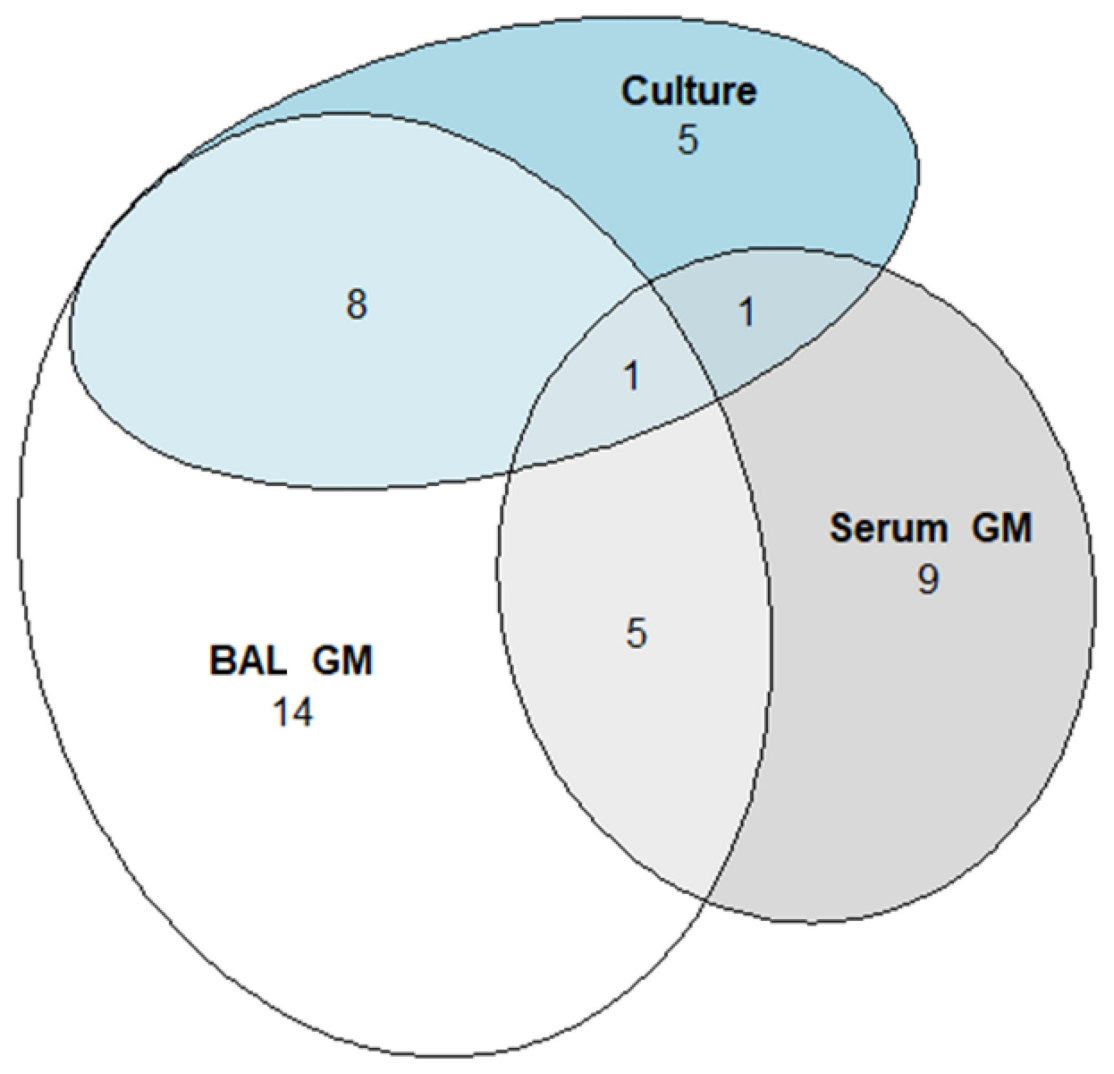
Venn diagram showing overlap in mycological criteria present in post-pathway patients diagnosed 382 with probable IFD. GM = galactomannan

In 89 episodes (18.5%) bronchoscopy was performed. Of the group proceeding to bronchoscopy, BAL PCR for Aspergillus was measured on 81 occasions (91.0%) and BAL galactomannan measured on 87 occasions (97.8%). When sent, BAL galactomannan was positive on 32 of 87 tests (36.8%), of which six tests were concordant with a positive serum galactomannan result. BAL Aspergillus PCR was positive on 26 of 81 tests (32.1%), of which 17 were concordant with other microbiological tests (five episodes with a concurrent positive BAL galactomannan, two episodes with a positive serum galactomannan, two episodes with positive cytology, eight episodes with a combination of two or more of these tests positive). Of nine positive BAL Aspergillus PCR results unsupported by galactomannan results or cytology, two had radiological changes consistent with IFD and were by our use of the 2008 criteria classed as possible IFD: by the 2019 EORTC/MSG definition represent they would represent probable IFD.(5,6) Overall, tests performed at bronchoscopy fulfilled either direct or indirect mycological criterion for the diagnosis of probable IFD by 2008 EORC/MSG criteria in 27 cases where serum galactomannan was negative or not performed (Table 3, Figure 1).(5)

There was no significant difference in whether an antifungal medication was prescribed at treatment dose, including empirical therapy for persistent fever and targeted therapy for IFD, during the admission after the introduction of the pathway (p=0.79), but the pattern of antifungal prescriptions was significantly different between the two groups (p=0.04) (Table 2). Some patients in both groups were prescribed multiple antifungal agents during their admission, but before introduction of the pathway a lower proportion of these - 31 of 38 cases (81.5%) - involved a narrowing of the antifungal spectrum, as compared to 60 of 61 cases (98.4%) after introduction of the pathway. This most frequently involved switching from L-Amb to voriconazole. Usage of voriconazole was also noted to increase after institution of the pathway (53 episodes [10.7%] pre-pathway vs 63 episodes [13.7%] post-pathway).

A post-hoc subanalysis including only patients deemed high-risk for developing IFD (acute leukaemia, MDS, and patients having undergone allogeneic HSCT) showed similar results to the full analysis. These patients had no significantly different risk of being prescribed at least one therapeutic antifungal after introduction of the pathway (p=0.37), but did have a different overall pattern of antifungal prescriptions (p=0.006), primarily due to increased narrowing of the antifungal spectrum after introduction of the pathway (Supplementary Table 1).

There was no difference in 30-day mortality (p=0.21) or 1-year mortality (p=0.57) following introduction of the pathway. A positive serum galactomannan was associated with a different overall distribution of antifungal medication prescriptions in comparison to the galactomannan-negative group (Table 3, p=0.001).

## Discussion

Our study showed that patterns of antifungal prescription were modified by a biomarker-informed approach to IFD diagnosis, suggesting that the use of biomarkers permits more targeted therapy in some cases.(7) In particular, increased use of biomarkers promoted more confident diagnosis of IFD contemporaneously, as evidenced by more frequent narrowing of the therapeutic spectrum to the most specific antifungal agent, voriconazole (Table 2). Given that a positive galactomannan test will fulfill a mycological criterion for the diagnosis of IFD, it is reassuring that, after introduction of the pathway, an increased proportion of cases could be designated as probable IFD (Table 2). Indeed, there was a particularly notable increase in the use of antifungal medications alongside more frequent diagnosis of IFD in the serum galactomannan-positive subset of 32 inpatient episodes (Table 3). Nonetheless, despite the increased diagnostic information provided by the use of biomarkers in the new pathway, there was no change to the overall proportion of patients who were prescribed an antifungal medication as empirical therapy for neutropaenic fever or targeted therapy for IFD.(11) Beyond alterations in therapy, our data demonstrated no difference in short or long-term survival after the introduction of biomarkers to the Trust, suggesting that incorporation of biomarkers in the diagnosis of IFD is safe though this was not the primary focus of the study (Table 1).

Importantly, of 320 episodes with a negative serum galactomannan test, 55 (17.2%) were deemed retrospectively to have possible or probable IFD and 86 (26.9%) episodes were of sufficient clinical concern to receive antifungal treatment, suggesting that in clinical practice a negative serum galactomannan result is sometimes not sufficient to withhold antifungal therapy (Table 3). Given that previous work calculated a sensitivity of 78% and specificity of 85% of the test for an ODI of >0.500, we would expect approximately 18 false negative tests in our prospective cohort (with an estimated IFD prevalence of 16.8%): in practice, it is clinically challenging to establish which patients represent these false negatives.(14) False positive results are also an issue with 50.0% of our positive serum galactomannan results not supported by radiological or other microbiological evidence and therefore classed as IFD-negative. Of five episodes including two distinct positive serum galactomannan results, three were classed as false positives. The reasons for false positive results are well-established and include enteral nutrition and antibiotics as well as, pertinently for this population, blood transfusion product components and myeloma itself.(15–17)

One reason for the disparity between previous analyses of serum galactomannan utility and our results could be real-life variation in antifungal prophylaxis. In the retrospective group at least one prophylactic antifungal agent was documented in 318 episodes (63.9%), with agents with anti-mould activity (i.e. excluding fluconazole) used in 105 episodes (21.1%). In the prospective group, an antifungal prophylactic agent was recorded in 382 episodes (79.3%), of which anti-mould agents constituted 205 episodes (42.5%). As anti-mould prophylaxis, but not fluconazole, decreases the sensitivity of the galactomannan assay, it is possible that greater use of these agents over time has led to false negative serum galactomannan results in our prospective cohort.(18,19) Other explanations for the lack of clinical utility of serum galactomannan could include incomplete availability of test results at the time of starting treatment, overtreatment (perhaps related to clinician anxiety) or deterioration prompting aggressive therapy. As such, our work would support the presence of AFS programmes and post-prescription review (particularly after negative biomarker results) as critical to the prevention of overtreatment in this patient group.(7)

Limitations intrinsic to this study include that it represents a single centre and its before-and-after design, which means that factors other than the new pathway could be responsible for the changes seen. Whilst the increase in 30-day mortality in the prospective cohort (12.0% vs 8.9% in the retrospective cohort) is surprising given improvements in outcomes for haematological malignancy over time, it is important to note that this change is not statistically significant and that 1 year mortality is slightly lower in the prospective group (36.4% vs 37.4%). We would therefore caution against over-interpretation of trends in the 30-day mortality data. Another explanation could be wider use of chemotherapy in a marginally older - and therefore higher risk - patient group over time, though this is beyond the scope of this study. As discussed, the EORTC/MSG guidelines are designed primarily for research rather than clinical use and results would be altered - though not significantly - by use of the 2019 EORTC/MSG guidelines which permit PCR results as positive mycological criteria for IFD diagnosis and a broader range of radiological changes as indicative of IFD. Finally, it was not possible to extract reliable data on timing of initiation of antifungal therapy relative to test result and length of antifungal therapy though this would add useful information to the study.

As this was a real-life study, not all patients in the prospective cohort underwent biomarker testing: this may reflect the difficulties in integrating a new pathway into clinical practice or clinician adaptation to a test perceived to be of limited utility. This is supported by the very infrequent use of antifungal medication and low rates of diagnosis of IFD in the subset of patients who did not undergo serum galactomannan testing (Table 3). That this subgroup avoided further investigation suggests that they were clinically well without ongoing pyrexia - as supported by the fact that cross-sectional imaging was performed in 31 of these episodes. Nonetheless, 75.5% (364/482) of the prospective cohort underwent at least one form of biomarker testing, therefore it is unlikely that complete concordance with the pathway would have demonstrated a significantly different result. The need for PCR testing to be performed at a reference laboratory distant to the Trust increased turnaround time and, as a result, significantly limited test uptake. Notably, the current NHS Prescribed Specialist Services (PSS) 1 Medicines Optimisation and Stewardship CQUIN (Commissioning for Quality and Innovation) Indicator incentivises all UK hospital Trusts to increase the use of on-site fungal diagnostics, aiming to reduce turnaround time, decrease costs, and, ultimately, improve antifungal stewardship.(20) Our work would support this aim, though locally we have found this difficult to achieve due to staff shortages and, more recently, the impacts of the COVID-19 pandemic.

Establishing a confident diagnosis of IFD in this patient group remains a significant challenge. In particular, it remains of critical importance to optimize the use and interpretation of biomarkers which increasingly form part of the diagnostic work-up, including as a part of pathways such as our own. Key components of decision making include risk assessment by experienced clinicians, antifungal stewardship with post-prescription review, and specialist thoracic radiology input.(7,21) Whilst our study did not show that the incorporation of biomarkers into clinical practice alone was sufficient to empower clinicians to withhold antifungal therapy, it is clear that they can play an important role in the multidisciplinary decision-making process and lead to a more confident and concerted narrowing of the therapeutic spectrum in selected cases. Future research - particularly the ongoing BioDriveAFS Trial - will aim to assess further whether the use of biomarkers for surveillance in high-risk haemato-oncology patients results in reduced antifungal prescription in a randomised, prospective setting.(22) Outside of further trials, it would also be useful pursue qualitative research on the factors driving prescription of antifungal medication in the absence of convincing evidence of IFD and how such behaviour could best be influenced. Ultimately, our centre’s experience highlights the challenges of interpreting fungal diagnostics in complex, real-world situations which include varying practice by treating clinicians and logistical difficulties with testing.

## Supplementary Material

Supplementary Figures and Table

## Figures and Tables

**Table 1:** Comparison of patients before and after introduction of a biomarker-inclusive pathway for management of febrile neutropaenia in haemato-oncology patients.

	Pre-pathway	Post-pathway
Total patients	302	308
Sex, n (%)		
Female	122 (40.4%)	134 (43.5%)
Male	180 (59.6%)	174 (56.5%)
Age range, y	15-85	16-89
Mean	52	56
Median	55	60
IQR	18 (44-62)	19 (48-67)
Haematological diagnosis, n (%) ALL		
ALL	26 (8.6%)	17 (5.5%)
AML	93 (30.8%)	103 (33.4%)
CLL	13 (4.3%)	14 (4.5%)
Lymphoma	102 (33.8%)	106 (34.4%)
MDS	17 (5.6%)	17 (5.5%)
MPN	5 (1.7%)	2 (0.6%)
Myeloma	36 (11.9%)	43 (14.0%)
Other	10 (3.3%)	6 (1.9%)
Haematopoetic Stem Cell Transplant, n (%) Allogeneic		
Allogeneic	75 (24.8%)	74 (24.0%)
Autologous	71 (23.5%)	71 (23.1%)
Both	1 (0.3%)	0 (0.0%)
None	155 (51.3%)	163 (52.9%)
Mortality, n (%)†		
30 days	27 (8.9%)	37 (12.0%)
1 year	113 (37.4%)	112 (36.4%)

† Mortality calculated from first day of final hospital admission.

**Table 2:** Investigation and management of episodes of febrile neutropaenia before and after introduction of the pathway.

	Pre-pathway	Post-pathway
Total inpatient episodes	497	482
Antifungal prophylaxis, n (%)† Fluconazole		
Fluconazole	240 (48.3%)	174 (36.1%)
Itraconazole	6 (1.2%)	93 (19.3%)
Posaconazole	0 (0.0%)	9 (1.9%)
Voriconazole	16 (3.2%)	36 (7.5%)
L-Amb	83 (16.7%)	67 (13.9%)
Micafungin	0 (0.0%)	1 (0.2%)
None	180 (36.2%)	103 (21.4%)
IFD diagnosis, n (%)		
No criteria	419 (84.3%)	401 (83.2%)
Possible	70 (14.1%)	38 (7.9%)
Probable	6 (1.2%)	43 (8.9%)
Proven	2 (0.4%)	0 (0.0%)
Treatment, n (%)		
No therapeutic antifungal	382 (76.9%)	367 (76.1%)
L-Amb only	48 (9.7%)	38 (7.9%)
Voriconazole only	21 (4.2%)	12 (2.5%)
L-Amb then voriconazole	25 (5.0%)	46 (9.5%)
Other combination therapy‡	13 (2.6%)	15 (3.1%)
Other therapeutic antifungal	8 (1.6%)	4 (0.8%)
Bronchoscopy performed, n (%)	66 (13.3%)	89 (18.5%)
CT-thorax performed, n (%)	262 (52.7%)	247 (51.2%)

† Figures sum to > number of inpatient episodes due to patients on multiple prophylactic agents.

‡ ≥2 of voriconazole, posaconazole, itraconazole, caspofungin, L-Amb given either sequentially or contemporaneously during the episode. ALL = acute lymphoblastic leukaemia; AML = acute myeloid leukaemia; CLL = chronic lymphocytic leukaemia; MDS = myelodysplastic syndrome; MPN = myeloproliferative neoplasm; IFD = invasive fungal disease.

**Table 3:** Impacts of serum galactomannan testing in patients admitted under a new pathway for febrile neutropaenia incorporating biomarkers.

	Serum galactomannan		
	Positive	Negative	Not performed
Total episodes	32	320	130
IFD diagnosis, n (%)			
No criteria	16 (50.0%)	265 (82.8%)	120 (92.3%)
Possible	0 (0.0%)	30 (9.4%)	8 (6.2%)
Probable	16 (50.0%)	25 (7.8%)	2 (1.5%)
Proven	0 (0.0 %)	0 (0.0%)	0 (0.0%)
Treatment, n (%)			
No therapeutic antifungal	15 (46.9%)	234 (73.1%)	118 (90.8%)
L-Amb	3 (9.4%)	30 (8.8%)	5 (3.9%)
Voriconazole	3 (9.4%)	7 (2.2%)	2 (1.5%)
L-Amb then voriconazole	10 (31.3%)	31 (9.7%)	5 (3.9%)
Other combination therapy†	1 (3.1%)	14 (4.4%)	0 (0.0%)
Other therapeutic antifungal	0 (0.0%)	4 (1.3%)	0 (0.0%)

† ≥2 of voriconazole, posaconazole, itraconazole, caspofungin, L-Amb given either sequentially or contemporaneously during the episode.

## References

[1] Enoch DA, Yang H, Aliyu SH, Micallef C (2017). The Changing Epidemiology of Invasive Fungal Infections. Methods Mol Biol.

[2] Corzo-León DE, Satlin MJ, Soave R, Shore TB, Schuetz AN, Jacobs SE, Walsh TJ (2015). Epidemiology and outcomes of invasive fungal infections in allogeneic haematopoietic stem cell transplant recipients in the era of antifungal prophylaxis: a single-centre study with focus on emerging pathogens. Mycoses.

[3] Herbrecht R, Caillot D, Cordonnier C, Auvrignon A, Thiébaut A, Brethon B, Michallet M, Mahlaoui N, Bertrand Y, Preziosi P, Ruiz F (2012). Indications and outcomes of antifungal therapy in French patients with haematological conditions or recipients of haematopoietic stem cell transplantation. J Antimicrob Chemother.

[4] Denning DW (2000). Early diagnosis of invasive aspergillosis. Lancet.

[5] De Pauw B, Walsh TJ, Donnelly JP, Stevens DA, Edwards JE, Calandra T, Pappas PG, Maertens J, Lortholary O, Kauffman CA, Denning DW (2008). Revised definitions of invasive fungal disease from the European Organization for Research and Treatment of Cancer/Invasive Fungal Infections Cooperative Group and the National Institute of Allergy and Infectious Diseases Mycoses Study Group (EORTC/MSG). Clin Infect Dis an Off Publ Infect Dis Soc Am.

[6] Donnelly JP, Chen SC, Kauffman CA, Steinbach WJ, Baddley JW, Verweij PE, Clancy CJ, Wingard JR, Lockhart SR, Groll AH, Sorrell TC (2020). Revision and Update of the Consensus Definitions of Invasive Fungal Disease From the European Organization for Research and Treatment of Cancer and the Mycoses Study Group Education and Research Consortium. Clin Infect Dis an Off Publ Infect Dis Soc Am.

[7] Micallef C, Aliyu SH, Santos R, Brown NM, Rosembert D, Enoch DA (2015). Introduction of an antifungal stewardship programme targeting high-cost antifungals at a tertiary hospital in Cambridge, England. J Antimicrob Chemother.

[8] White PL, Linton CJ, Perry MD, Johnson EM, Barnes RA (2006). The evolution and evaluation of a whole blood polymerase chain reaction assay for the detection of invasive aspergillosis in hematology patients in a routine clinical setting. Clin Infect Dis an Off Publ Infect Dis Soc Am.

[9] Barnes RA, White PL, Bygrave C, Evans N, Healy B, Kell J (2009). Clinical impact of enhanced diagnosis of invasive fungal disease in high-risk haematology and stem cell transplant patients. J Clin Pathol.

[10] Barnes R, Earnshaw S, Herbrecht R, Morrissey O, Slavin M, Bow E, McDade C, Charbonneau C, Weinstein D, Kantecki M, Schlamm H (2015). Economic Comparison of an Empirical Versus Diagnostic-Driven Strategy for Treating Invasive Fungal Disease in Immunocompromised Patients. Clin Ther.

[11] Morrissey CO, Chen SC-A, Sorrell TC, Milliken S, Bardy PG, Bradstock KF, Szer J, Halliday CL, Gilroy NM, Moore J, Schwarer AP (2013). Galactomannan and PCR versus culture and histology for directing use of antifungal treatment for invasive aspergillosis in high-risk haematology patients: a randomised controlled trial. Lancet Infect Dis.

[12] Martinelli AW, Patil P, Wong VK, Enoch DA, Sander CR (2019). A positive BAL galactomannan in non-haemato-oncology patients risks harmful overtreatment. J Med Microbiol.

[13] Matthey F, Parker A, Rule SAJ, Wimperis JZ, Ardeshna KM, Bird JM, Cullis J, Lyttelton MPA, McMillan A, Jackson GH (2010). Facilities for the treatment of adults with haematological malignancies--’Levels of Care’: BCSH Haemato-Oncology Task Force 2009. Hematology.

[14] Leeflang MMG, Debets-Ossenkopp YJ, Wang J, Visser CE, Scholten R, Hooft L, Bijlmer HA, Reitsma JB, Zhang M, Bossuyt PMM (2015). Galactomannan detection for invasive aspergillosis in immunocompromised patients. Cochrane Database Syst Rev.

[15] Ansorg R, van den Boom R, Rath PM (1997). Detection of Aspergillus galactomannan antigen in foods and antibiotics. Mycoses.

[16] Martín-Rabadán P, Gijón P, Alonso Fernández R, Ballesteros M, Anguita J, Bouza E (2012). False-positive Aspergillus Antigenemia Due to Blood Product Conditioning Fluids. Clin Infect Dis.

[17] Mori Y, Nagasaki Y, Kamezaki K, Takenaka K, Iwasaki H, Harada N, Miyamoto T, Abe Y, Shimono N, Akashi K, Teshima T (2010). High incidence of false-positive Aspergillus galactomannan test in multiple myeloma. American journal of hematology.

[18] Marr KA, Laverdiere M, Gugel A, Leisenring W (2005). Antifungal therapy decreases sensitivity of the Aspergillus galactomannan enzyme immunoassay. Clin Infect Dis an Off Publ Infect Dis Soc Am.

[19] Cornely OA (2014). Galactomannan testing during mold-active prophylaxis. Clinical infectious diseases : an official publication of the Infectious Diseases Society of America.

[20] Heafield S, Qualie M (2020). PSS1 Medicines Optimisation and Stewardship PSS CQUIN Indicator.

[21] Agrawal S, Barnes R, Brüggemann RJ, Rautemaa-Richardson R, Warris A (2016). The role of the multidisciplinary team in antifungal stewardship. J Antimicrob Chemother.

[22] Barlow G, Allsup D, Sheard L, Hilton A, Lillie P, Tharmanathan P, Ali S, Corbacho B, Fairhurst C, Torgerson D, Hope W (2021). Biomarker Driven Antifungal Stewardship (BioDriveAFS) in Acute Leukaemia a Multi-Centre Randomised Controlled Trial to Assess Clinical and Cost Effectiveness. NIHR Funding and Awards.

