# Supplementary material for: Introducing biomarkers for invasive fungal disease in haemato-oncology patients: a single-centre experience
Source: J Med Microbiol. Author manuscript; Available in PMC 2022 Jul 28. (PMC7613179; doi:10.1099/jmm.0.001564)
Supplement: Supplementary Figures and Table [file EMS150533-supplement-Supplementary_Figures_and_Table.pdf]

Supplementary Material

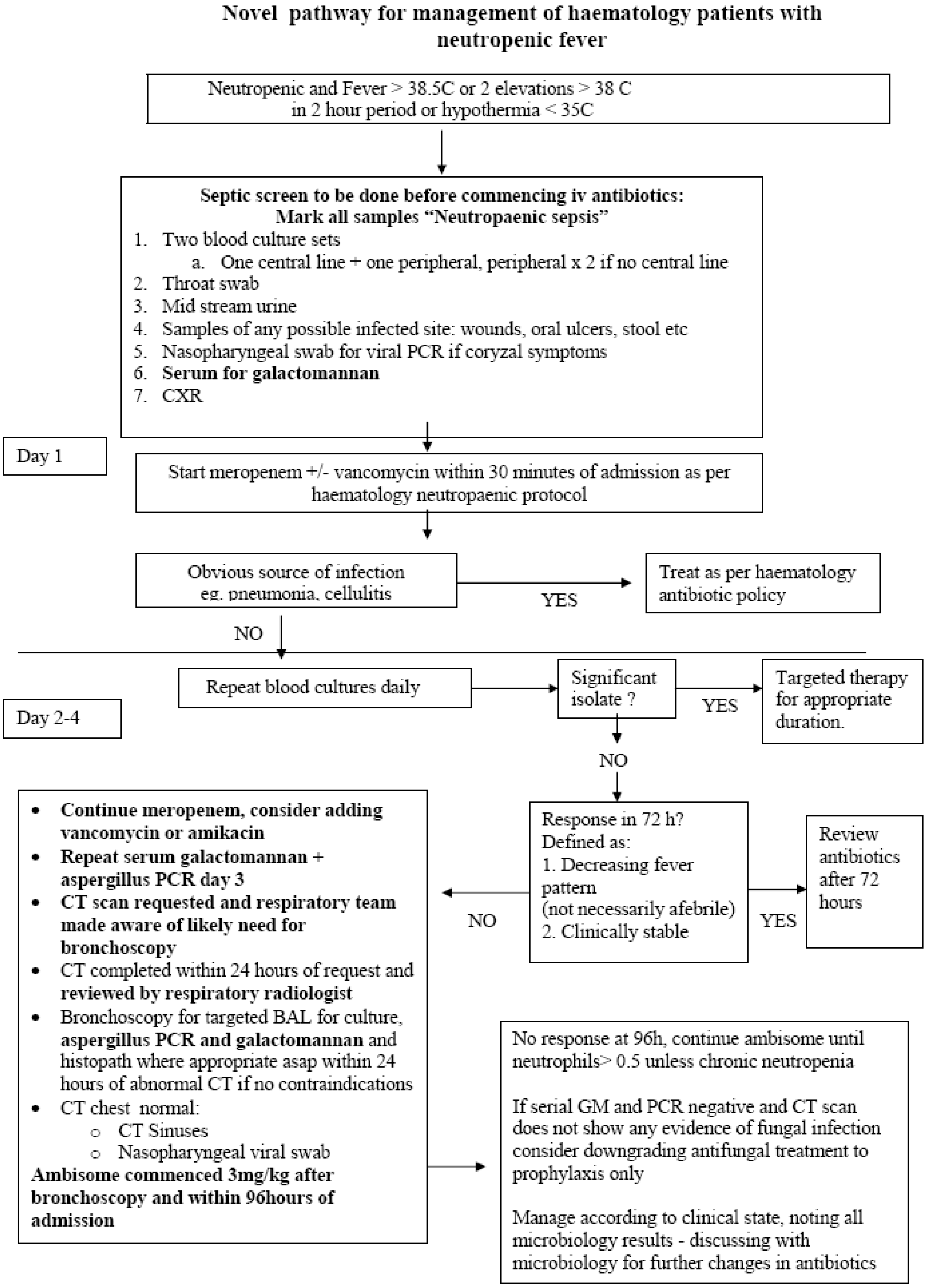

Supplementary Figure 1 *Novel pathway for management of haematology patients with neutropaenic fever.*

A

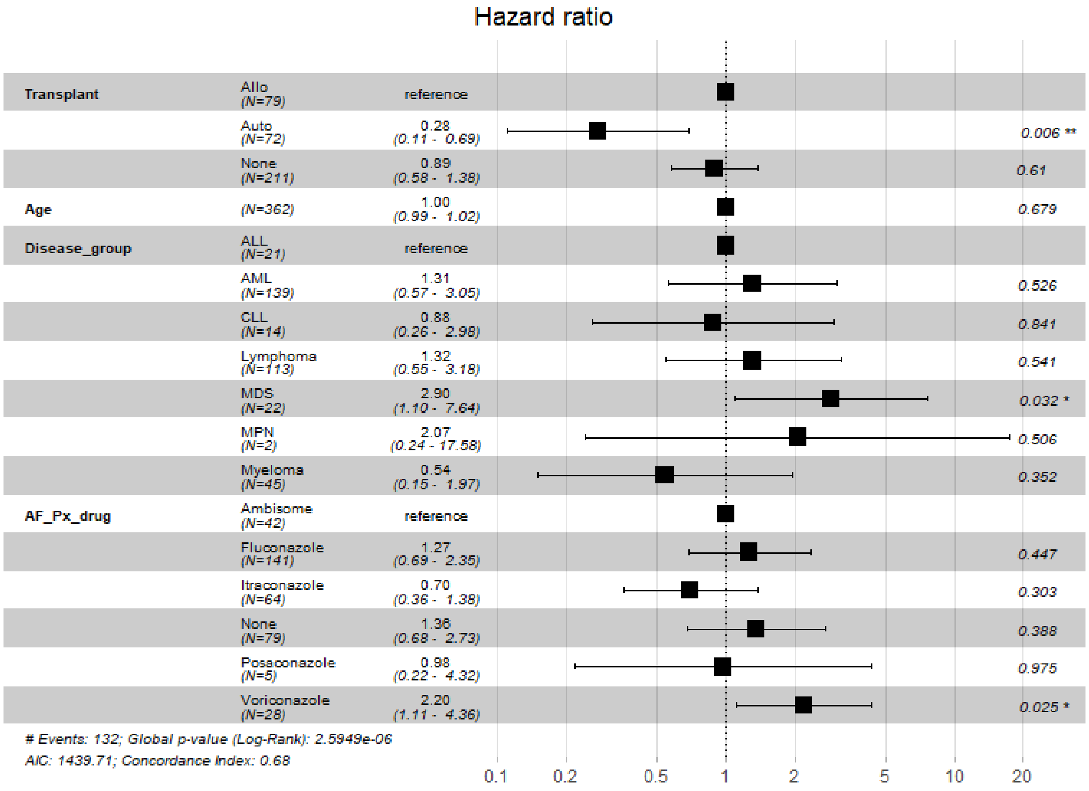

B

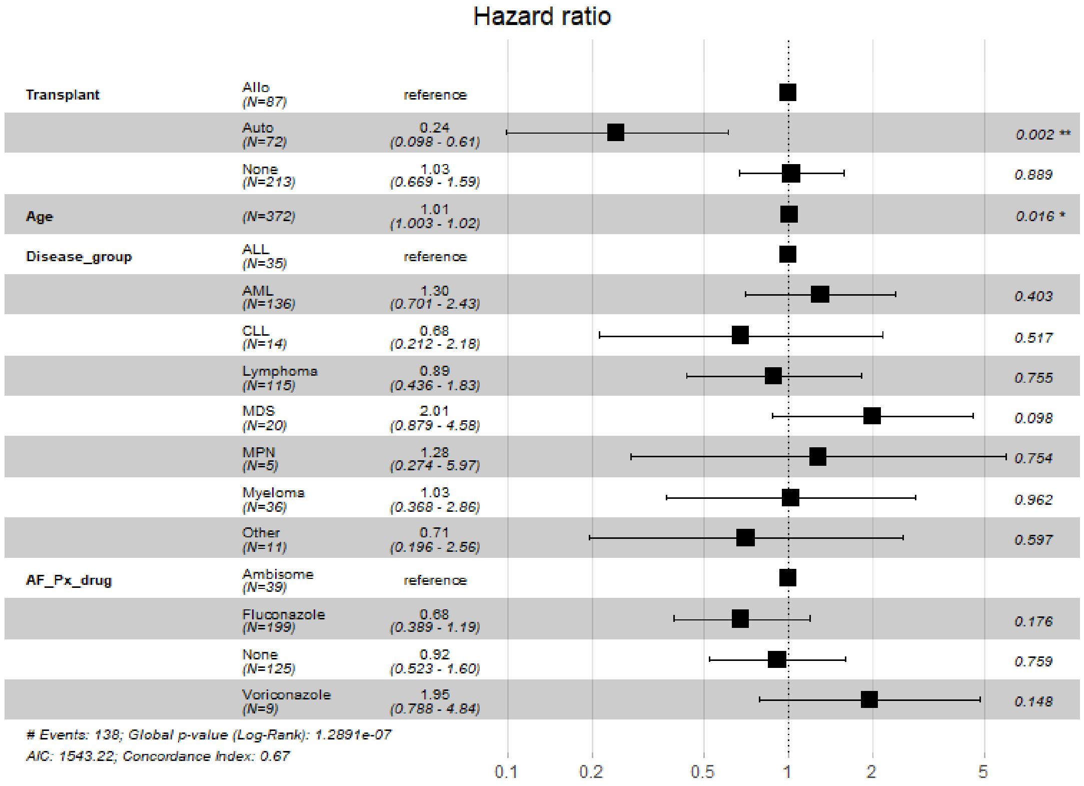

Supplementary Figure 2 *Cox proportional hazards models for predictors of overall survival at 1 year in prospective (A) and retrospective (B) patient cohorts.*

	**Pre-pathway**	**Post-pathway**
Total patients	171	159
Sex, n (%)		
Female	60 (35.1%)	68 (42.7%)
Male	111 (64.9%)	91 (57.2%)
Age range, y	16-85	16-89
Mean	49	54
Median	54	56
IQR	21 (41-61)	20 (46-65)
Haematological diagnosis, n (%)		
ALL	26 (15.2%)	17 (10.7%)
AML	93 (54.4%)	103 (64.8%)
CLL	7 (4.1%)	5 (3.1%)
Lymphoma	16 (9.3%)	14 (8.8%)
MDS	17 (9.9%)	17 (10.7%)
MPN	5 (2.9%)	1 (0.6%)
Myeloma	3 (1.8%)	1 (0.6%)
Other	4 (2.3%)	1 (0.6%)
Haematopoetic Stem Cell Transplant, n (%)		
Allogeneic	75 (43.9%)	74 (46.5%)
Autologous	2 (1.2%)	0 (0.0%)
Both	1 (0.6%)	0 (0.0%)
None	93 (54.4%)	85 (53.5%)
Mortality, n (%)†		
30 days	22 (12.9%)	20 (12.6%)
1 year	82 (47.9%)	73 (45.9%)
Total inpatient episodes	327	299
Antifungal prophylaxis, n (%)§		
Fluconazole	146 (44.6%)	76 (25.4%)
Itraconazole	6 (1.8%)	89 (29.8%)
Posaconazole	0 (0.0%)	9 (3.0%)
Voriconazole	16 (4.9%)	34 (11.4%)
L-Amb	74 (22.7%)	64 (21.4%)
Micafungin	0 (0.0%)	1 (0.3%)
None	109 (33.3%)	27 (9.0%)
IFD diagnosis, n (%)		
No criteria	267 (81.7%)	236 (78.9%)
Possible	56 (17.1%)	29 (9.7%)
Probable	3 (0.9%)	34 (11.4%)
Proven	1 (0.3%)	0 (0.0%)
Treatment, n (%)		
No therapeutic antifungal	237 (72.5%)	207 (69.2%)
L-Amb only	37 (11.3%)	25 (8.4%)
Voriconazole only	16 (4.9%)	9 (3.0%)
L-Amb then voriconazole	20 (6.1%)	45 (15.1%)
Other combination therapy‡	10 (3.1%)	10 (3.3%)
Other therapeutic antifungal	7 (2.1%)	3 (1.0%)

† Mortality calculated from first day of final hospital admission.
§ Figures sum to > number of inpatient episodes due to patients on multiple prophylactic agents.
‡ ≥2 of voriconazole, posaconazole, itraconazole, caspofungin, L-Amb given either sequentially or contemporaneously during the episode.

ALL = acute lymphoblastic leukaemia; AML = acute myeloid leukaemia; CLL = chronic lymphocytic leukaemia; MDS = myelodysplastic syndrome; MPN = myeloproliferative neoplasm; IFD = invasive fungal disease.

Supplementary Table 1 *Comparison of patients with high-risk pathology before and after introduction of a biomarker-inclusive pathway for management of febrile neutropaenia in haemato-oncology patients.*
